# Analytic Morphomics in Myositis-Related Interstitial Lung Disease

**DOI:** 10.1007/s00408-023-00637-3

**Published:** 2023-07-17

**Authors:** Alexander T. O’Mahony, Patrick J. Henry, Patrick Coghlan, Michael Waldron, Claire Crowley, David Ryan, Niamh Moore, Deirdre M. Bennett, Owen J. O’Connor, Michael M. Maher, Michael T. Henry

**Affiliations:** 1 Department of Radiology, Cork University Hospital, Cork, Ireland; 2 School of Medicine, University College Cork, Cork, Ireland; 3 Department of Respiratory Medicine, Cork University Hospital, Cork, Ireland; 4 Department of Radiology, University College Cork, Cork, Ireland; 5 Department of Radiology, Mercy University Hospital, Cork, Ireland; 6 Department of Radiography, School of Medicine, University College Cork, Cork, Ireland

**Keywords:** Idiopathic inflammatory myopathies, Interstitial lung disease, Analytic morphomics, Body composition

## Abstract

**Purpose:**

Interstitial lung disease (ILD) is the most common non-musculoskeletal manifestation of idiopathic inflammatory myopathies (IIM). Identification of body composition change may enable early intervention to improve prognosis. We investigated muscle quantity and quality derived from cross-sectional imaging in IIM, and its relationship to ILD severity.

**Methods:**

A retrospective cohort study assessing IIM of ILD patients (n = 31) was conducted. Two datasets separated in time were collected, containing demographics, biochemical data, pulmonary function testing and thoracic CT data. Morphomic analysis of muscle quantity (cross-sectional area) and quality (density in Hounsfield Units) on thoracic CT were analysed utilising a web-based tool allowing segmentation of muscle and fat. Bilateral erector spinae and pectoralis muscle (ESM&PM) were measured at defined vertebral levels.

**Results:**

FVC and D_L_CO decreased but within acceptable limits of treatment response (FVC: 83.7–78.7%, p < 0.05, D_L_CO 63.4–60.6%, p < 0.05). The cross-sectional area of the PM and ESM increased (PM: 39.8 to 40.7 cm^2^, p = 0.491; ESM: 35.2 to 39.5 cm^2^, p = 0.098). Density significantly fell for both the PM and ESM (PM: 35.3–31 HU, p < 0.05; ESM: 38–33.7, p < 0.05). Subcutaneous fat area increased from 103.9 to 136.1 cm^2^ (p < 0.05), while the visceral fat area increased but not reaching statistical significance. The change in PM density between time points demonstrated an inverse correlation with D_L_CO (p < 0.05, R =  − 0.49).

**Conclusion:**

Patients with IIM ILD demonstrated significant body composition changes on CT imaging unlikely to be detected by traditional measurement tools. An increase in muscle area with an inverse decrease in density suggests poor muscle quality.

**Supplementary Information:**

The online version contains supplementary material available at 10.1007/s00408-023-00637-3.

## Introduction

Idiopathic inflammatory myopathies (IIM) are a heterogenous group of disorders encompassing polymyositis, dermatomyositis, clinically amyopathic dermatomyositis and immune mediated necrotising myopathies [1]. Various classification criteria have been previously proposed, culminating in those recently published by the European League Against Rheumatism [2–6].

The hallmark of lung involvement in patients with IIM is the presence of interstitial lung disease (ILD), present in approximately 30% and preceding muscular symptoms in 20% of cases [7, 8]. Despite advancements in treatment, ILD still has a median survival of 5–7 years [9–11]. Changes in body composition, such as sarcopenia (decreased muscle mass and strength) and myosteatosis (muscle fat infiltration) have been linked to adverse outcomes in patients with various pulmonary and non-pulmonary diseases. Specifically, in lung cancer, its been shown that these changes can lead to an increase in post-operative complications and hospital stays [12–14], as well as a heightened risk of ILD exacerbation in non-small cell lung cancer in those undergoing chemotherapy treatment [15]. Additionally, sarcopenia has been shown to have a significant correlation with COPD severity [16], however its relationship with idiopathic pulmonary fibrosis (IPF) is less clear [17, 18]. Reduced pectoral muscle area has been shown on CT thorax imaging to correlate with FVC and DLCO in ILD, supporting the hypothesis that ILD progression is associated with sarcopenia [23]. Additionally, the presence of sarcopenia on clinical assessment been shown to be associated with disease progression in IPF patients [24, 25]. In many extra-thoracic conditions these markers are emerging as key prognostic factors related to a heterogenous range of systemic disease processes [19–22].

Traditional body composition and nutritional status assessment methods include dual-energy x-ray absorptiometry, air displacement plethysmography, bioelectric impedance analysis. Recently, magnetic resonance imaging (MRI) and computed tomography (CT) have been utilised [26]. Analytic morphomics, which encompass quantitative metrics from cross-sectional imaging can offer insights into organ health, muscle quality, fat distribution, and bone measures including mineral density [19]. Quantitative data can be extracted using validated segmentation software (e.g. CoreSlicer). This software uses manual, semi-automated or fully automated image segmentation software algorithms that rely on differences in attenuation values (Hounsfield units, HU) between structures [20, 21, 27]. This analysis method termed threshold-based segmentation a new approach to body composition assessment. The European Working Group on Sarcopenia in Older People (EWGSOP) recently suggested it potential for widespread future use due to its strong correlation with whole body muscle [28]. Hence, using analytic morphomics from routine thoracic CT scans to assess patients with IIM related ILD could potentially enable early detection of body composition changes, facilitating interventions to preserve or enhance muscle mass and thereby improve overall prognosis.

This study aims to evaluate body composition in IIM related ILD patients using analytic morphomics from clinically indicated thoracic CT scans. We hypothesise a correlation between thoracic muscle cross-sectional area (CSA) and muscle density, lung function and patient outcomes. If validated, this approach could enable earlier intervention to preserve or enhance muscle mass in myositis related ILD patients, potentially, improving their prognosis.

## Methods

### Study Type

Single centre retrospective study, conducted in accordance with institutional review board ethical approval (Reference number: ECM 4 (j) 09/02/2021).

### Demographics

Patients with a known diagnosis of IIM-related ILD confirmed at a regional ILD multidisciplinary meeting, attending a single regional ILD centre for specialist management between 2014 and 2020 were included. Thirty-one patients were included in the final analysis (n = 31). In our study, we assessed a cohort of patients diagnosed with idiopathic inflammatory myopathies (IIM), with diagnosis confirmed through clinical evaluations and, if necessary, biopsies. Given the cohort size limitations and the diverse range of myositis subtypes and associated antibodies within our cohort, we opted to focus on IIM as a collective group, instead of conducting subtype-based analyses, to ensure the statistical robustness and reliability of our findings.

### Clinical Data

Data obtained from our institutional IIM database included patient demographics, smoking status, baseline (t = 0) bloods (renal function, creatinine kinase (CK), c-reactive protein (CRP), albumin, white cell count (WCC) and alanine transaminase (ALT)), pulmonary function tests (forced vital capacity (FVC) and single breath carbon monoxide gas transfer factor (D_L_CO) both expressed as absolute values and percent predicted normal), CT morphometrics (see below), myositis antibody status and treatment received during the study period. A further set of data was collected and analysed (t = 1) after an interval, median 17 months (IQR 12–22). The interval time was recorded as duration between CT scans with biochemistry and pulmonary function testing occurring within 3 months of scanning (t = 0 or t = 1).

### CT Morphometrics

Morphometric analysis was performed on previously acquired CT imaging of the thorax. CT images were acquired using 64-slice multidetector row CT scanners (GE Medical Systems Discovery CT 750HD, GE Medical Systems Lightspeed VCT, GE Medical discovery STE). The scan parameters were: tube voltage of 120 kVp, automated tube current modulation (ATCM) 50–400 mA. Of the 62 individual scans analysed, 42 received intravenous contrast (Iohexol, Omnipaque 300, GE Healthcare, Waukesha, WI, USA), 9 of these were dedicated CT pulmonary angiograms and 4 were as part of cumulative thorax, abdomen, and pelvic imaging. All included studies had a slice acquisition of 1.25 mm independent of scan protocol used. Scans that did not include the entire thorax or with inadequate resolution were excluded. Scans of the thorax were assessed by 2 independent pulmonary radiologists with a specialist interest in ILD.

**Fig. 1:**
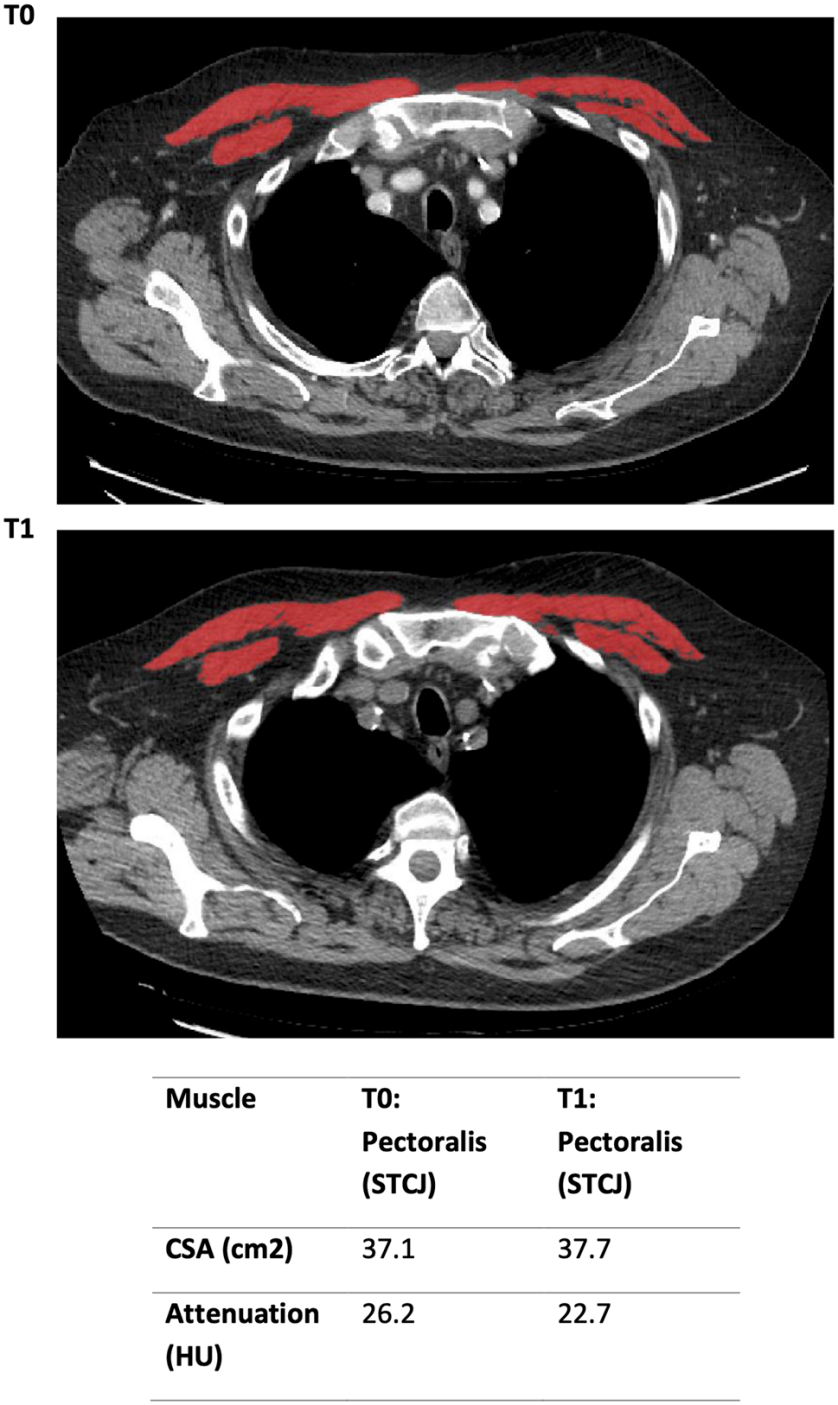
Cross-sectional area of the pectoralis muscle within 1 cm of the sternoclavicular joint (STCJ) at t = 0 and t = 1

**Fig. 2:**
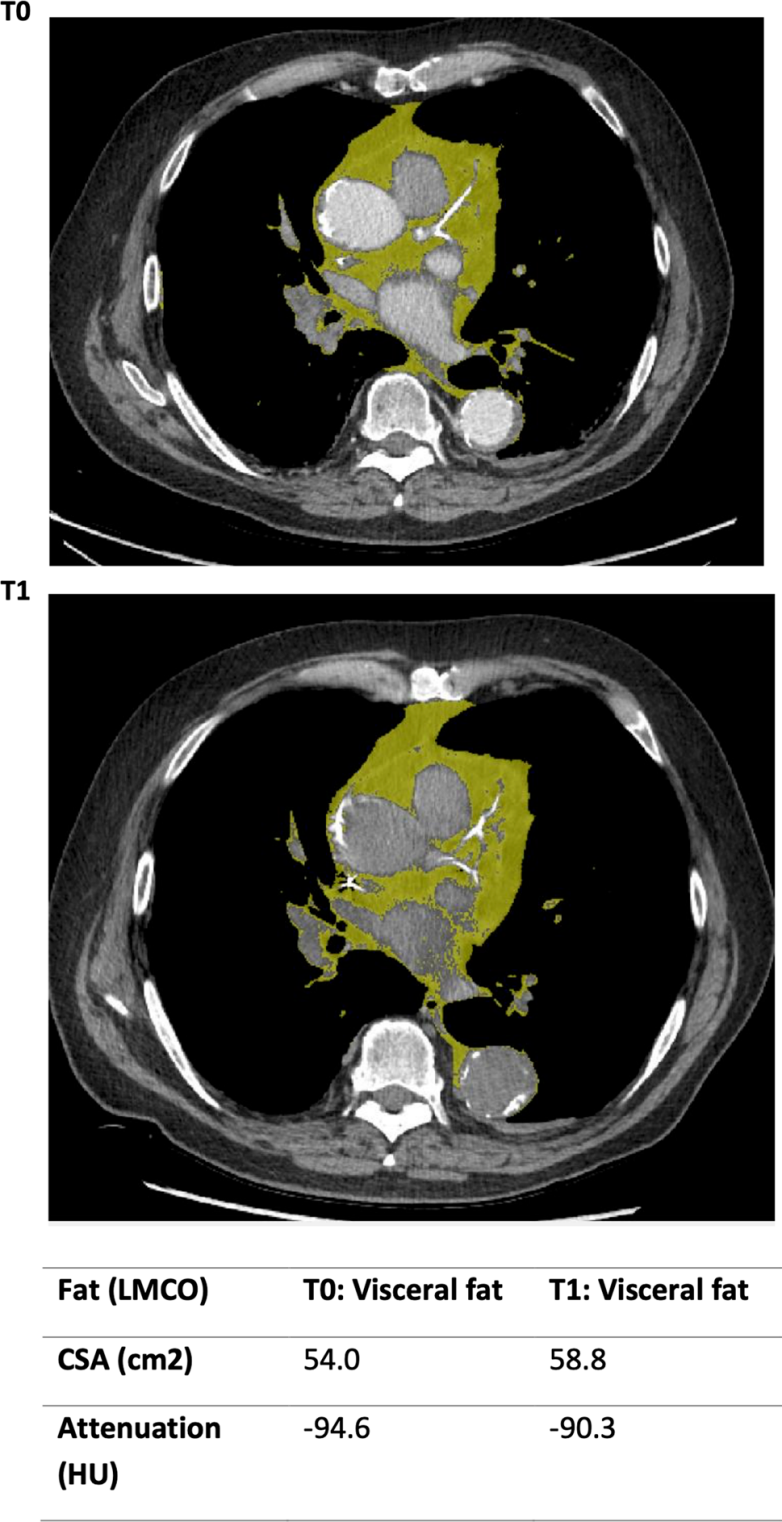
Cross-sectional area of visceral mediastinal fat at the left main coronary artery outflow at t = 0 and t = 1

**Fig. 3:**
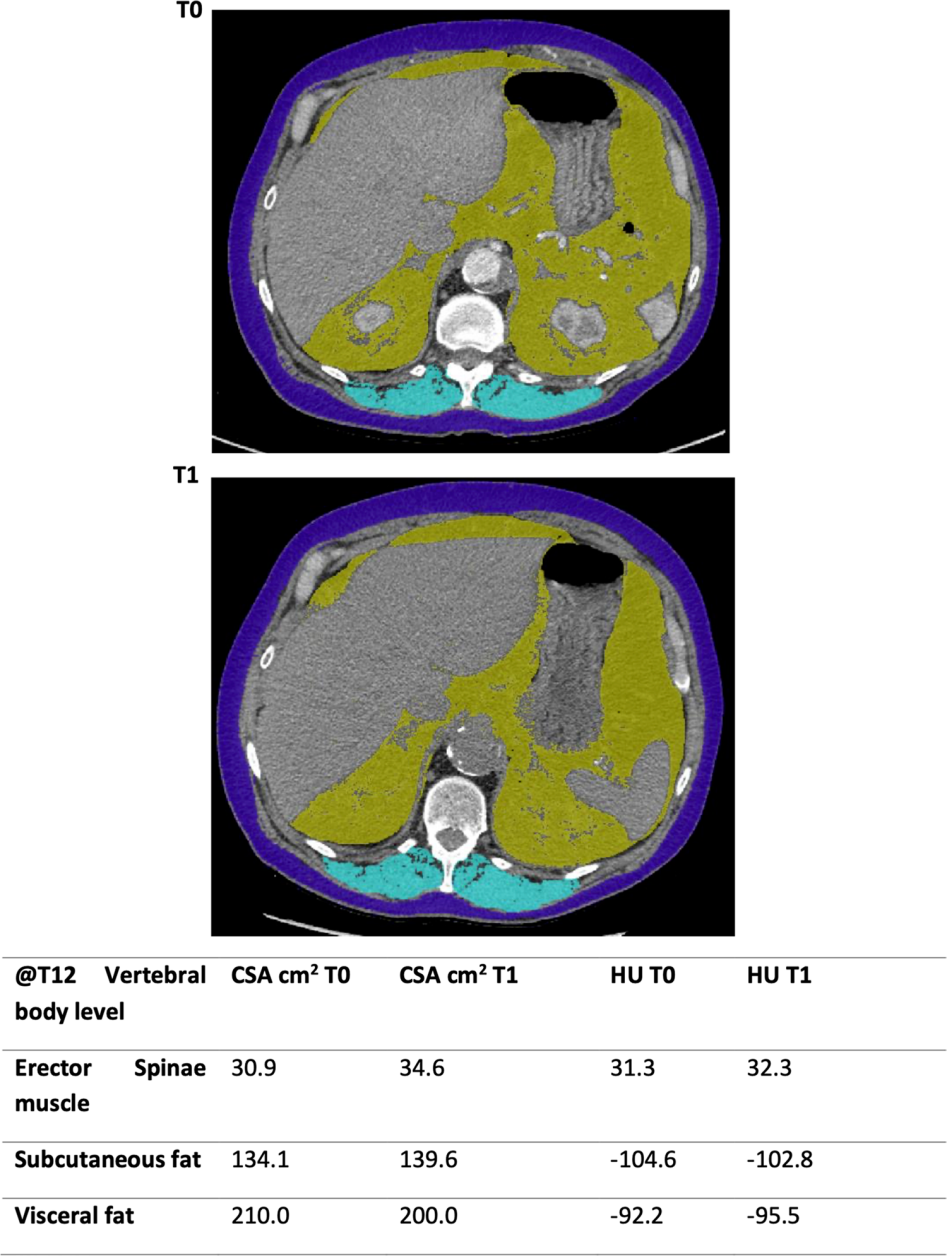
The cross-sectional area (CSA) and attenuation (HU) of erector spinae muscle (ESM), subcutaneous and visceral fat at the level of the 12th thoracic vertebrae superior end plate at t = 0 and t = 1

Images were analysed using CoreSlicer, a web-based tool that enables semi-automated segmentation of muscle (− 29 to + 150 HU) and fat (− 190 to − 30 HU) [27]. On a single axial slice at the level of the lower margin of the 12th thoracic vertebra erector spinae muscle (ESM), subcutaneous and visceral fat cross-sectional area (CSA) and average attenuation in HU were calculated. A further single axial slice of within 1 cm of the sternoclavicular joints was used to calculate these parameters for pectoralis muscle (PM) and finally an axial slice at the level of the left main coronary artery outflow (LMCO) for visceral fat cross-sectional area and HU. This process was repeated for both thoracic CT scans for each patient. CSA was further adjusted for height resulting in an index for muscle or fat described as cm^2^/m^2^ (Figs. 1, 2, 3).

### Statistical Analysis

Data compilation and statistical analysis were performed using Microsoft Excel 2011 (Microsoft Corporation, Washington, USA) and Statistical Package for the Social Sciences (SPSS) version 28 (IBM, Chicago, Illinois, USA). Wilcoxon signed-rank test was used to compare median values for non-parametric data. A p-value of < 0.05 was considered significant. Z values were calculated. The critical Z score values using a 95% confidence interval are − 1.96 and + 1.96 standard deviations.

Univariate analysis was used to determine the correlation between clinical and morphomic variables and lung function. Pearson or Spearman correlation co-efficient was used to assess for correlation between morphometrics and clinical parameters. Interval change in morphometrics and clinical parameters was also assessed by Pearson or Spearman correlation co-efficient.

## Results

### Patient Demographics and Radiological Findings

Thirty-one patients diagnosed with IIM between 2014 and 2020 were identified. Fifty-two percent (n = 16) were male. Median age was 72 years (IQR 64–78). Median BMI 28.7 (IQR 25.7–32.6). Sixty-five percent (n = 20) were ex-smokers, 32% (n = 10) were non-smokers. There was one current smoker. Six (19%) patients died during the treatment period (Supplementary Appendix 1).

Patient treatment regimens included rituximab n = 17, corticosteroids n = 13, mycophenolate mofetil n = 5, nintedanib, pirfenidone, tacrolimus, tofacitinib and cyclophosphamide n = 1 each. Fourteen patients received a combination of > 1 of the treatments listed above, and 4 patients required no treatment. Patient antibody profiles included anti-Ro-52 antibody n = 9, anti-PL-12 n = 5, anti-PM/Sc l75 n = 4, anti-Ku n = 4, anti-PL-7 n = 3, anti-SRP n = 3, anti-Jo-1 n = 2, anti-Mi-2 beta n = 2, anti-MDA-5 n = 1 and anti-TIF-1-gamma n = 1. Findings suggestive of pulmonary hypertension were noted in 4 patients on T1 CT thorax. We observed varied ILD patterns of lung parenchyma affected. There were 22 patients (73.3%) who demonstrated a pattern of lower lobe predominance, five patients (16.7%) presented with upper lobe predominance, a less commonly observed pattern in this patient population. Additionally, a subset of 4 patients (13.3%) exhibited diffuse lung involvement, signifying a more extensive disease process. These findings underscore the heterogeneity of lung involvement in IIM-associated ILD and the importance of detailed radiographic evaluation in this patient population.

### Clinical Indices

Table 1 below summarises measured indices at or closest to initial thoracic CT scanning (T0) and at follow-up imaging (T1). The median duration between index and interval was 17 months (IQR 12–22).

**Table 1:** Summary of clinical indices at T0 and T1

	T0 Index			T1 Interval			Wilcoxon Rank	
	Median	IQR Low	IQR High	Median	IQR Low	IQR High	P-value	Z
Clinical biochemistry								
White cell count (× 10^9^/L)	7.6	6.2	11.2	7.6	6.3	10.0	0.594	
C-reactive protein (mg/L)	8.8	2.2	19.6	5.0	2.9	11.3	0.026	− 2.22
Albumin (g/L)	41.0	36.0	42.0	40.0	38.0	42.0	0.282	
Creatinine (μmol/L)	83.0	67.0	94.3	89.0	77.0	99.0	0.008	− 2.67
Urea (mmol/L)	6.6	4.8	7.7	6.6	5.5	8.5	0.290	
Creatinine kinase (U/L)	114.0	59.0	263.0	96.0	65.3	160.5	0.009	− 2.62
Alanine transaminase (ALT) (U/L)	25.6	17.4	31.0	28.0	21.4	33.1	0.214	
Spirometry								
FVC% Predicted	83.7	73.5	105.9	78.7	66.6	93.4	0.002	− 3.12
DLCO% Predicted	63.4	46.0	83.5	60.6	42.3	76.5	0.008	− 2.66
Morphometrics								
Muscle								
Pectoralis CSA in cm^2^	39.8	28.8	52.2	40.7	30.5	50.9	0.491	
Pectoralis HU	35.3	29.4	40.9	31.0	27.4	38.3	0.035	− 2.11
Pectoralis Index (CSA/m^2^)	14.3	11.0	16.7	14.4	11.7	18.8	0.453	
Erector spinae CSA in cm^2^	35.2	28.7	41.0	39.5	31.3	43.3	0.098	
Erector spinae HU	38.0	32.2	46.1	33.7	27.4	40.4	0.023	− 2.27
Erector spinae index (CSA/m^2^)	12.4	10.5	14.2	13.6	11.8	15.7	0.094	
Fat								
Subcutaneous CSA T12 in cm^2^	103.9	87.0	205.8	136.1	93.9	195.9	0.009	− 2.60
Subcutaneous Index (CSA/m^2^)	36.9	29.5	77.9	44.7	32.3	76.2	0.009	− 2.62
Visceral CSA T12 in cm^2^	110.4	61.7	180.6	128.1	69.3	187.6	0.178	
Visceral Index (CSA/m^2^)	43.7	22.7	60.1	44.4	25.6	62.8	0.237	
Visceral CSA LMCO in cm^2^	23.2	19.7	33.8	27.4	19.2	37.2	0.147	
Visceral: Subcutaneous Ratio	0.9	0.5	1.7	0.8	0.6	1.8	0.926	

Characteristics of patients with IIM measured at diagnosis before treatment (T = 0) and after a variable period of treatment (T = 1). Blood parameters, lung function and morphometric data including muscle cross sectional area and density are included. Data are expressed as medians with interquartile ranges

*IQR* interquartile range, *FVC% predicted* forced vital capacity expressed as percent predicted, *DLCO% predicted* single breath carbon monoxide gas transfer factor expressed as percent predicted, *Pectoralis CSA* pectoralis muscle cross sectional area expressed as centimetres squared, *Erectors spinae CSA* erector spinae muscle cross sectional area expressed as centimetres, *Pectoralis HU* pectoralis muscle density expressed as Hounsfield units, *Erector spinae HU* erector spinae muscle density expressed as Hounsfield units, *Pectoralis index* pectoralis muscle cross-sectional area in centimetres squared divided by height squared in metres, *Erector spinae index* erector spinae muscle cross-sectional area in centimetres squared divided by height squared in metres

The CRP and CK fell significantly during the study period. Included objective measures of pulmonary function as represented by percentage predicted also decreased. The FVC at T0 fell from 83.7 to 78.7% at T1. The D_L_CO from 63.4 to 60.6%, p = 0.002, 0.008 respectively.

As indicated by CSA there was a net gain in measured PM and ESM from T0 to T1. The PM area at T0 was 39.8 cm^2^ (28.8–52.2) with a PM index (PMI = CSA in cm^2^/height in m^2^) of 14.3 cm^2^/m^2^ (11–16.7) increasing to a PM of 40.7 cm^2^ (30.5–50.9) and PMI of 14.4 cm^2^/m^2^ (11.7–18.8) at T1. The ESM area and ESM index (ESMI) at T0 of 35.2 cm^2^ (28.7–41) and 12.4 cm^2^/m^2^ (10.5–14.2) also increased to an area of 39.5 cm^2^ (31.3–43.3) and ESMI of 13.6 cm^2^/m^2^ (11.8–15.7). However, neither of these changes reached statistical significance.

In contrast to increasing PM and ESM size, there was a notable decrease in the attenuation, for PM density a reduction from 35.3 HU (29.4–40.9) to 31 HU (27.4–38.3), p < 0.05 occurred. In ESM density, a reduction from 38 HU (32.2- 46.1) to 33.7 HU (27.4–40.4), p = 0.023 occurred.

There appears to be an increased level of subcutaneous and visceral fat measured at the T12 vertebral level over the study interval. The subcutaneous fat increased from 130.9 to 136.1 cm^2^ (p < 0.05) and visceral fat from 110.1 to 128.1 cm^2^ although the latter did not reach significance (p = 0.178). The increasing regional fat distribution over the interval occurred with a preserved visceral-to-subcutaneous fat ratio (0.9 at T0, 0.8 at T1), p = 0.926.

### Clinical Correlation

Table 2 below summarises the bivariate analyses (Pearson and Spearman correlation coefficient) for the change in indices measured between the two timepoints (T0 & T1) over the study duration. For example, the change in PM density over the study interval showed a relatively moderate inverse correlation with change in D_L_CO (p < 0.05, R =  − 0.49). Albumin and the ESM area showed a linear relationship and changed together over the interval (p < 0.05, R = 0.562).

**Table 2:** Correlation of clinical metrics with change in CT derived morphometrics

Correlation matrix	*∆FVC*	*∆DLCO*	*∆WCC*	*∆CRP*	*∆Albumin*	*∆Creatinine*	*∆Urea*	*∆CK*	*∆ALT*
∆Pectoralis CSA in cm^2^	0.560*	0.962*	0.254*	0.335*	0.581*	0.560*	0.360*	0.301*	0.411*
∆Pectoralis HU ND	0.787	0.01 (R = − 0.49)	0.626*	0.205*	0.102	0.901*	0.958	0.957*	0.867*
∆Erector spinae CSA in cm^2^ ND	0.532	0.656	0.543*	0.257*	0.003 (R = 0.562)	0.267*	0.184	0.304*	0.342*
∆Erector spinae HU	0.921*	0.673*	0.121*	0.417*	0.478*	0.724*	0.299*	0.529*	0.571*
∆Subcutaneous CSA T12 in cm^2^	0.437*	0.432*	0.645*	0.171*	0.995*	0.911*	0.858*	0.954*	0.896*
∆Visceral CSA T12 in cm^2^	0.899*	0.757*	0.308*	0.269*	0.348*	*0.939*	0.527*	0.697*	0.417*
∆Visceral CSA LMCO in cm^2^	0.255	0.335	0.166*	0.896*	0.293	0.718*	0.630	0.868*	0.810*

This table represents the change in the specified variable between T0 and T1. Pearson correlation was carried out when assessing the delta of two variables with normal distribution. Otherwise, the Spearman coefficient was used. Use of Spearmans test was indicated by a *. Values indicated are p-values and where a p-value < 0.05 and corresponding R-value

In Supplementary Appendix 2 the static correlation between morphometrics at T0 and T1 with pulmonary function testing is summarised. PM density correlated with FVC (p = 0.034, R = 0.396) and visceral mediastinal fat CSA with D_L_CO inversely (p = 0.045, R =  − 0.389).

### Subgroup Analysis: Steroid Exposure

Although no statistically significant change occurred in the analytic morphomics of those in the study when categorised by steroid exposure a number of differences appear to exist. The PM area of those not exposed to steroid treatment demonstrated a net gain with a median of + 1.01 cm^2^ (− 4.03 to + 5.70) and a modest decrease in attenuation − 2 HU (− 7.88 to + 3.12) vs. a net loss with a median of − 0.06 cm^2^ (− 3.67 to + 4.5) and a higher loss in attenuation, − 5.98 HU (− 10.82 to + 0.45) in the steroid exposed group. Both groups had increasing SCF area and VF area over the study period but those with steroid exposure had a larger increase in the VF area (6.57 vs. 24.37, p = 0.49), although not significant (Table 3).

**Table 3:** Summary of differences in change in analytic morphomics for steroid exposure over the study interval

Subgroup: corticosteroid exposure	No steroid treatment	IQR Min	IQR Max	Steroid treatment	IQR Min	IQR Max	P-value
Change (T0–T1)							
*∆*Pectoralis CSA in cm^2^	1.01	− 4.03	5.70	− 0.06	− 3.67	4.50	0.691
*∆*Pectoralis HU	− 2.00	− 7.88	3.12	− 5.98	− 10.82	0.45	0.127
*∆*Erector spinae CSA in cm^2^	1.97	− 3.90	7.88	3.72	− 1.59	8.06	0.464
*∆*Erector spinae HU	− 4.42	− 8.62	1.85	− 2.93	− 8.70	4.59	0.786
*∆*Subcutaneous fat CSA T12 in cm^2^	11.02	− 5.62	22.14	15.04	− 0.84	29.01	0.325
*∆*Visceral fat CSA T12 in cm^2^	6.57	− 23.96	34.02	24.37	− 6.41	45.12	0.49
*∆*Visceral fat CSA LMCO in cm^2^	2.69	− 0.61	13.27	− 0.10	− 11.49	8.83	0.187

This table represents the change in the specified variable between T0 and T1 represented as median change with interquartile range and variable metric according to parameter specification. The p-value relates to a Wilcoxon Ranks test conducted between the median values of the two groups

## Discussion

The use of threshold based segmental tools in cross-sectional imaging and the derivation of quantitative metrics role in prognosis has shown great potential. Specifically, in ILD, and the IPF subtype, a decrease in muscle CSA can potentially predict future risk of mortality. In this similar group, a decline in the ESM CSA correlated with decline in FVC and ultimately earlier mortality [29].

Prognostic factors for IIM include presence of poor lung function associated with ILD at diagnosis, which is predictive of long term deterioration [30, 31]. This study investigated isolated muscle area and density at predefined levels in IIM, focusing on its relationship to ILD disease severity, and possible role in prognosis and demonstrated that muscle density falls over time in a group of heterogenous IIM patients with no corresponding decrease in muscle CSA. We suggest that this is due to myosteatosis, a replacement of muscle tissue with additional fat, which does not appear to be related to ongoing systemic inflammation. Inflammatory markers such as WCC, CK and CRP levels fell in this group, with treatment presumably, over time. The FVC decreased by on average 5% and DLCO by 2.8%, these are modest and considered acceptable indicating success in a cohort exposed to treatment as described by Wells 2013 in Idiopathic Pulmonary Fibrosis (IPF) [32].

The hallmark of lung involvement in IIM is the presence of ILD, where it is present in at a third of patients [33, 34] and preceding muscular symptoms in about 20% of cases [35]. The presentation of patients with myositis associated ILD can be categorised into two distinct clinical patterns, namely rapidly progressive ILD, and the more common chronic ILD that is slowly progressive [36]. Lung function test results were indicative of restrictive lung patterns of ILD in IIM patients, reinforced the diagnosis when combined with other clinical findings. The current study demonstrates a modest lung function decline over the treatment period in the analysed group. A novel finding in this study was the role of muscle attenuation in IIM ILD as opposed to the CSA and a preservation of muscle CSA may be in fact a false reassurance. The change in PM HU demonstrated an inverse correlation with the change in the DLCO over the treatment period. Albeit counterintuitive, this is potentially explained by declining DLCO causing a compensatory maintenance of the PM composite make-up and maintaining its role as an accessory muscle. Thus, explaining the finding not being mirrored in the ESM HU. More intuitively the linear correlation of albumin and ESM CSA over the study interval alludes to the use of muscle metrics from CT capable of assessing nutritional status.

Several studies have looked at the effect of treatment on sarcopenia in other connective tissues diseases demonstrating that after administration of glucocorticoid therapy for a year, 13.4% of the rheumatoid arthritis patients developed sarcopenia with a OR of 8.81 (95% CI 1.146–7.9, p = 0.037) [37]. The results imply that glucocorticoid treatment should be used cautiously and that reduction or stopping steroids could alleviate treatment-related sarcopenia [38]. There was an apparent greater increase in both visceral and subcutaneous fat area in the steroid group than their counterparts with a more pronounced effect in the visceral component. This may represent the fat redistribution effects of glucocorticoids with its central preponderance as previously described elsewhere [39]. However, within this sub analysis the effects of steroids on muscle were more dynamic and difficult to interpret most likely due to the small sample analysed.

The present study has the following limitations. The results were obtained from a single tertiary referral centre by retrospective analysis. The cohort size was small and as such the subtypes of IIM based on the myositis associated antibodies and treatment administered precluded subgroup analysis. Further studies in a larger replication cohort will be required to validate our observations. While the most prominent muscle enzymes were evaluated, levels of certain markers such as aldolase, AST, and lactate dehydrogenase were not assessed routinely in all patients. There is no international consensus for diagnosis of IIM associated ILD, so diagnosis was based on local guidelines and MDT diagnosis with treatment based on international consensus documents. We were unable to adjust for survival analysis as most patients survived throughout the treatment and follow up period. Small sample size precluded multivariate cox regression analysis for comparison of muscle CSA and density analysis with multiple clinical variables.

## Conclusion

Quantitative metrics assessing body composition using threshold based segmental tools derived from routine cross-sectional imaging may have a distinct role in IIM ILD, a cohort requiring routine thoracic CT. In this group, muscle CSA of the pectoralis and erector spinae muscle groups is preserved over time but with a significant change in the attenuation i.e., quality not quantity shift. This change in attenuation of the PM has an inverse relationship with the D_L_CO. Fat redistribution and increased risk of sarcopenia is a possibility in those with glucocorticoid exposure, but a larger sample size is required for more definitive analysis.

## Supplementary Information

Below is the link to the electronic supplementary material.

Supplementary file1 (DOCX 14 KB)

Supplementary file2 (DOCX 15 KB)

## Abbreviations

BMI: Body mass index
COPD: Chronic obstructive pulmonary disease
CT: Computed tomography
CK: Creatinine kinase
CRP: C-reactive protein
CSA: Cross-sectional area
D_L_CO: Diffusing capacity of lung for carbon monoxide
ESM: Erector spinae muscle
ESMI: Erector spinae muscle index
FVC: Forced vital capacity
HU: Hounsfield Units
IIM: Idiopathic inflammatory myopathy
IPF: Idiopathic pulmonary fibrosis
ILD: Interstitial lung disease
LMCO: Left main coronary artery outflow
PM: Pectoralis muscle
PMI: Pectoralis muscle index
PFTs: Pulmonary function tests
SCF: Subcutaneous fat
MRI: Magnetic resonance imaging
MHC: Major histocompatibility complex
MDT: Multidisciplinary team
VF: Visceral fat
WCC: White cell count

## References

[1] Mecoli CA, Christopher-Stine L (2018). Management of interstitial lung disease in patients with myositis specific autoantibodies. Curr Rheumatol Rep.

[2] McHugh NJ, Tansley SL (2018). Autoantibodies in myositis. Nat Rev Rheumatol.

[3] Medsger TA, Dawson WN, Masi AT (1970). The epidemiology of polymyositis. Am J Med.

[4] Bohan A, Peter JB (1975). Polymyositis and dermatomyositis (first of two parts). N Engl J Med.

[5] Bohan A, Peter JB (1975). Polymyositis and dermatomyositis (second of two parts). N Engl J Med.

[6] Malaviya AN (2018). 2017 EULAR/ACR classification criteria for adult and juvenile idiopathic inflammatory myopathies and their major subgroups: little emphasis on autoantibodies, why?. Ann Rheum Dis.

[7] Kuwana M, Gil-Vila A, Selva-O’Callaghan A (2021). Role of autoantibodies in the diagnosis and prognosis of interstitial lung disease in autoimmune rheumatic disorders. Therap Adv Musculoskelet Dis.

[8] Selva-O’Callaghan A, Romero-Bueno F, Trallero-Araguás E (2021). Pharmacologic treatment of anti-MDA5 rapidly progressive interstitial lung disease. Curr Treat Opt Rheumatol.

[9] Douglas WW, Tazelaar HD, Hartman TE (2001). Polymyositis-dermatomyositis-associated interstitial lung disease. Am J Respir Crit Care Med.

[10] Nakashima R, Hosono Y, Mimori T (2016). Clinical significance and new detection system of autoantibodies in myositis with interstitial lung disease. Lupus.

[11] Mandel D, Malemud C, Askari A (2017). Idiopathic inflammatory myopathies: a review of the classification and impact of pathogenesis. IJMS.

[12] Degens JHRJ, Sanders KJC, de Jong EEC (2019). The prognostic value of early onset, CT derived loss of muscle and adipose tissue during chemotherapy in metastatic non-small cell lung cancer. Lung Cancer.

[13] Kawaguchi Y, Hanaoka J, Ohshio Y (2021). Does sarcopenia affect postoperative short- and long-term outcomes in patients with lung cancer?—a systematic review and meta-analysis. J Thorac Dis.

[14] Anjanappa M, Corden M, Green A (2020). Sarcopenia in cancer: risking more than muscle loss. Tech Innov Patient Support Radiat Oncol.

[15] Kikuchi R, Takoi H, Ishiwari M (2022). Impact of sarcopenia on chemotherapy-triggered exacerbation of interstitial lung disease in patients with non-small cell lung cancer. Thorac Cancer.

[16] Moon SW, Choi JS, Lee SH (2019). Thoracic skeletal muscle quantification: low muscle mass is related with worse prognosis in idiopathic pulmonary fibrosis patients. Respir Res.

[17] Cortellini A, Verna L, Porzio G (2019). Predictive value of skeletal muscle mass for immunotherapy with nivolumab in non-small cell lung cancer patients: a “hypothesis-generator” preliminary report. Thoracic Cancer.

[18] Tanimura K, Sato S, Fuseya Y (2016). Quantitative assessment of erector spinae muscles in patients with chronic obstructive pulmonary disease novel chest computed tomography-derived index for prognosis. Ann Am Thorac Soc.

[19] Stidham RW, Waljee AK, Day NM (2015). Body fat composition assessment using analytic morphomics predicts infectious complications after bowel resection in Crohn’s disease. Inflamm Bowel Dis.

[20] Zuckerman J, Ades M, Mullie L (2017). Psoas muscle area and length of stay in older adults undergoing cardiac operations. Ann Thorac Surg.

[21] Drudi LM, Phung K, Ades M (2016). Psoas muscle area predicts all-cause mortality after endovascular and open aortic aneurysm repair. Eur J Vasc Endovasc Surg.

[22] Abbass T, Dolan R, McSorley ST (2020). Skeletal muscle index (SMI) status and survival in patients undergoing surgery for colorectal cancer (CRC): a longitudinal study. J Clin Oncol.

[23] Molgat-Seon Y, Guler SA, Peters CM (2021). Pectoralis muscle area and its association with indices of disease severity in interstitial lung disease. Respir Med.

[24] Faverio P, Fumagalli A, Conti S (2022). Sarcopenia in idiopathic pulmonary fibrosis: a prospective study exploring prevalence, associated factors and diagnostic approach. Respir Res.

[25] Fujita K, Ohkubo H, Nakano A (2022). Frequency and impact on clinical outcomes of sarcopenia in patients with idiopathic pulmonary fibrosis. Chron Respir Dis.

[26] Calella P, Valerio G, Brodlie M (2018). Cystic fibrosis, body composition, and health outcomes: a systematic review. Nutrition.

[27] Mullie L, Afilalo J (2019). CoreSlicer: a web toolkit for analytic morphomics. BMC Med Imaging.

[28] Cruz-Jentoft AJ, Bahat G, Bauer J (2019). Sarcopenia: revised European consensus on definition and diagnosis. Age Ageing.

[29] Nakano A, Ohkubo H, Taniguchi H (2020). Early decrease in erector spinae muscle area and future risk of mortality in idiopathic pulmonary fibrosis. Sci Rep.

[30] Fujisawa T, Hozumi H, Kono M (2017). Predictive factors for long-term outcome in polymyositis/dermatomyositis-associated interstitial lung diseases. Respir Investig.

[31] Fujisawa T, Hozumi H, Kono M (2014). Prognostic factors for myositis-associated interstitial lung disease. PLoS ONE.

[32] Wells AU (2013). Forced vital capacity as a primary end point in idiopathic pulmonary fibrosis treatment trials: making a silk purse from a sow’s ear. Thorax.

[33] Barba T, Fort R, Cottin V (2019). Treatment of idiopathic inflammatory myositis associated interstitial lung disease: a systematic review and meta-analysis. Autoimmun Rev.

[34] Connors GR, Christopher-Stine L, Oddis CV, Danoff SK (2010). Interstitial lung disease associated with the idiopathic inflammatory myopathies: what progress has been made in the past 35 years?. Chest.

[35] Hervier B, Devilliers H, Stanciu R (2012). Hierarchical cluster and survival analyses of antisynthetase syndrome: phenotype and outcome are correlated with anti-tRNA synthetase antibody specificity. Autoimmun Rev.

[36] Ha YJ, Lee YJ, Kang EH (2018). Lung involvements in rheumatic diseases: update on the epidemiology, pathogenesis, clinical features, and treatment. Biomed Res Int.

[37] Yamada Y, Tada M, Mandai K (2020). Glucocorticoid use is an independent risk factor for developing sarcopenia in patients with rheumatoid arthritis: from the CHIKARA study. Clin Rheumatol.

[38] Fenton CG, Webster JM, Martin CS (2019). Therapeutic glucocorticoids prevent bone loss but drive muscle wasting when administered in chronic polyarthritis. Arthritis Res Ther.

[39] Lee MJ, Pramyothin P, Karastergiou K, Fried SK (2014). Deconstructing the roles of glucocorticoids in adipose tissue biology and the development of central obesity. Biochim Biophys Acta.

