# Supplementary material for: Analytic Morphomics in Myositis-Related Interstitial Lung Disease
Source: Lung. 2023 Jul 17;201(4):345–53. doi: 10.1007/s00408-023-00637-3 (PMC10444650; doi:10.1007/s00408-023-00637-3)
Supplement: Supplementary file 1 — Supplementary file1 (DOCX 14 KB) [file 408_2023_637_MOESM1_ESM.docx]

## Appendix S1

## Patient demographic summary

| Demographics | n=31 |  | IQR |
| --- | --- | --- | --- |
| Age |  | 72 | 64-78 |
| Gender | Male | 16 |  |
|  | Female | 15 |  |
| Smoking status | Current | 1 |  |
|  | Never | 10 |  |
|  | Previous | 20 |  |
| BMI (kg/m^2^) |  | 28.7 | 25.7-32.6 |
