# Supplementary material for: Analytic Morphomics in Myositis-Related Interstitial Lung Disease
Source: Lung. 2023 Jul 17;201(4):345–53. doi: 10.1007/s00408-023-00637-3 (PMC10444650; doi:10.1007/s00408-023-00637-3)
Supplement: Supplementary file 2 — Supplementary file2 (DOCX 15 KB) [file 408_2023_637_MOESM2_ESM.docx]

## Appendix S2

## Static Correlation of Height adjusted Morphometrics with Pulmonary Function Tests at T0 and T1.

| **T0** | | **PMI (cm^2^/m^2^)** | **ESMI (cm^2^/m^2^)** | **SCFI (cm^2^/m^2^)** | **VFI (cm^2^/m^2^)** | **VMFI (cm^2^/m^2^)** | **PM HU** | **ESM HU** |
| --- | --- | --- | --- | --- | --- | --- | --- | --- |
| **FVC%** | *Sig* | .054 | *.615 | *.095 | .116 | *.573 | .840 | .857 |
|  | *R-value* | N/A | N/A | N/A | N/A | N/A | N/A | N/A |
| **DLCO%** | *Sig* | .869 | *.631 | *.457 | .918 | *.737 | .702 | .663 |
|  | *R-value* | N/A | N/A | N/A | N/A | N/A | N/A | N/A |

| **T1** | | **PMI (cm^2^/m^2^)** | **ESMI (cm^2^/m^2^)** | **SCFI (cm^2^/m^2^)** | **VFI (cm^2^/m^2^)** | **VMFI (cm^2^/m^2^)** | **PM HU** | **ESM HU** |
| --- | --- | --- | --- | --- | --- | --- | --- | --- |
| **FVC%** | *Sig* | .085 | .066 | .696 | .190 | .819 | .034 | *.595 |
|  | *R-value* | N/A | N/A | N/A | N/A | N/A | 0.396 | N/A |
| **DLCO%** | *Sig* | 1.000 | .133 | .561 | .454 | .045 | .837 | *.817 |
|  | *R-value* | N/A | N/A | N/A | N/A | -0.389 | N/A | N/A |

Table Legend: PMI, pectoralis muscle index= pectoralis muscle area in cm squared/height in metres squared; ESMI, erector spinae muscle index= erector spinae muscle area in cm squared/height in metres squared; SCFI, subcutaneous fat index= subcutaneous fat area in cm squared/height in metres squared; VFI, visceral fat index= visceral fat area at T12 vertebral body in cm squared/height in metres squared; VMFI, visceral mediastinal fat area at the left main coronary artery outflow in cm squared/height in metres squared.
